# Impact of state telehealth policies on telehealth use among patients with newly diagnosed cancer

**DOI:** 10.1093/jncics/pkad072

**Published:** 2023-09-15

**Authors:** Tina W F Yen, I-Wen Pan, Ya-Chen Tina Shih

**Affiliations:** Department of Surgery, Division of Surgical Oncology, Medical College of Wisconsin, Milwaukee, WI, USA; Department of Health Services Research, The University of Texas MD Anderson Cancer Center, Houston, TX, USA; Department of Health Services Research, The University of Texas MD Anderson Cancer Center, Houston, TX, USA; Program in Cancer Health Economics Research, Jonsson Comprehensive Cancer Center, University of California Los Angeles, Los Angeles, CA, USA; Department of Radiation Oncology, David Geffen School of Medicine, University of California Los Angeles, Los Angeles, CA, USA

## Abstract

**Background:**

Telehealth restrictions were relaxed under the COVID-19 public health emergency. We examined telehealth use before and during the pandemic among patients with newly diagnosed cancers and the association between state policies and telehealth use.

**Methods:**

The study cohort was constructed from Optum’s deidentified Clinformatics Data Mart and included patients with lymphoma, female breast cancer, colorectal cancer, prostate cancer, and lung cancer diagnosed between March 1, 2019, and March 31, 2021. We performed an interrupted time series analysis to examine the trend of cancer-related telehealth use within 1 month of diagnosis relative to the timing of the COVID-19 public health emergency and multivariable logistic regressions to examine factors—specifically, state parity laws and regulations on cross-state practice—associated with telehealth.

**Results:**

Of 110 461 patients, the rate of telehealth use peaked at 33.4% in April 2020, then decreased to 12% to 15% between September 2020 and March 2021. Among the 53 982 patients diagnosed since March 2020, telehealth use was statistically significantly lower for privately insured patients residing in states with coverage-only parity or no or unspecified parity than those in states with coverage and payment parity (adjusted rate = 20.2%, 19.1%, and 23.3%, respectively). The adjusted rate was lower for patients in states with cross-state telehealth policy limitations than for those in states without restrictions (14.9% vs 17.8%).

**Conclusions:**

Telehealth use by patients diagnosed with cancer during the pandemic was higher among those living in states with more generous parity and less restrictive rules for cross-state practice. Policy makers contemplating whether to permanently relax certain telehealth policies must consider the impact on vulnerable patient populations who can benefit from telehealth.

Before the COVID-19 pandemic, the use of telehealth services to provide patient care was limited by laws and regulations that determined who, what, where, by whom, and in what fashion services can be delivered and how they will be reimbursed (1-3). The federal government regulates reimbursement and coverage of telehealth services for Medicare and self-insured plans. Medicaid and fully insured private health plans are regulated by federal and state policies, with tremendous variation in coverage and reimbursement for telehealth services across states. In fall 2019, 43 states and the District of Columbia had telehealth laws in place, 41 states and the District of Columbia mandated coverage parity (ie, require that the same services be covered whether in person or by telehealth), while only 22 states mandated both coverage parity and payment parity, which requires reimbursement of telehealth services on the same basis or at the same rate as in-person care) (4,5). Nine states issued licenses that allowed an out-of-state health-care professional to render telehealth services; 29 states and the District of Columbia had interstate medical licensure compact (IMLC) agreements, which streamline the licensing process for physicians who wish to practice in multiple states (6,7).

The COVID-19 public health emergency, declared January 31, 2020, resulted in the Centers for Medicare & Medicaid Services relaxing many telehealth restrictions, including allowing clinicians to practice across state lines and patients to access services from any location at any geographic region, waiving the need for a preexisting patient-clinician relationship, expanding telehealth coverage to additional services, and qualifying phone visits as telehealth visits (1-3,8). In addition, all states made temporary changes to telehealth policies, most expanded coverage of telehealth services, and most temporarily waived out-of-state licensing requirements (9,10). These changes resulted in a rapid adoption and substantial increase in telehealth use for general medical and oncology care in winter and spring 2020, with subsequent leveling but still higher use than prepandemic rates since summer 2020 (11-14).

Although the federal public health emergency ended on May 11, 2023, such declarations had already expired in most states. Many of the temporary licensing regulations began to lapse by mid-2021 (15). Because state governments decide which temporary changes to telehealth policies should stay, it is imperative to understand how state telehealth policies addressing coverage and payment parity and out-of-state or interstate licensing regulations are associated with telehealth use.

Telehealth is especially beneficial for patients who have travel limitations or require long-distance travel to receive care. For patients with cancer, this is particularly important because 20% live in rural areas, while only 7% of oncologists practice in these areas (16). Greater travel burden is associated with delays in cancer diagnosis, more advanced disease, more limited treatment options, lower receipt and quality of care, and higher cancer-related mortality (16-19). We therefore sought to examine the association between state telehealth policies and telehealth service use among patients with newly diagnosed cancer during the first year of the pandemic, when regulations regarding coverage, payment, and state licensing were the least restrictive.

## Methods

### Data source

This study cohort was constructed from Optum’s deidentified Clinformatics Data Mart database from December 2018 to June 2021. The database, comprising enrollees in commercial and Medicare Advantage plans, includes enrollment records as well as medical and pharmacy claims for members of a large insurance company with coverage spanning all 50 states plus the District of Columbia. The University of Texas MD Anderson Cancer Center’s Institutional Review Board determined that this study was exempt from formal review because it used deidentified data.

### Study cohort

We applied a previously published algorithm (20), using *International Statistical Classification of Diseases, Tenth Revision* diagnosis and procedure codes to identify incident cohorts of patients diagnosed with any of 5 common cancers—breast (female), prostate, lung, colorectal, and lymphoma—between March 2019 and March 2021 (Supplementary Table 1, available online). Inclusion criteria were 1) 3 or more cancer claims on separate dates within 3 months after the first date of diagnosis (index date) and 2) 6 months of full enrollment, 3 months before and after the index date. Patients with any cancer diagnosis within 3 months before the index date but who were missing state and age information were excluded. Finally, we created 2 study cohorts. The first included patients diagnosed with cancer between March 2019 and March 2021 to examine the trend of telehealth use before and during the pandemic. The second cohort included patients diagnosed after March 2020 to examine the association between state policies and telehealth use (Supplementary Table 1, available online).

### Outcomes

We used *Current Procedural Terminology and Healthcare Common Procedure Coding System* codes to identify telehealth use, including telehealth visits, e-visits, virtual check-in, or other temporary *Healthcare Common Procedure Coding System* codes for the COVID-19–related public health emergency, with a telehealth modifier “95,” “GT,” “GQ,” or a place of service code “02” (21). Telehealth use was defined as a telehealth claim with an associated cancer diagnosis within 1 month of cancer diagnosis (Supplementary Table 2, available online).

### State policies

State policies regarding telehealth for private health plans were extracted from National Conference of State Legislatures, Federation of State Medical Boards, and state executive orders (Supplementary Table 3, available online) in effect during the study period (4,22). State coverage and payment parity status was determined based on policies in effect before or during the public health emergency. Coverage parity requires that the same services delivered in person be covered for telehealth. Payment parity requires that the same payment rate or amount be reimbursed for care regardless of whether that care is provided through telehealth or delivered in person (5). State policies regarding cross-state telehealth were determined based on orders in effect during the 1-year study period. There were no known temporal changes to cross-state telehealth policies during this period.

**Figure 1.**
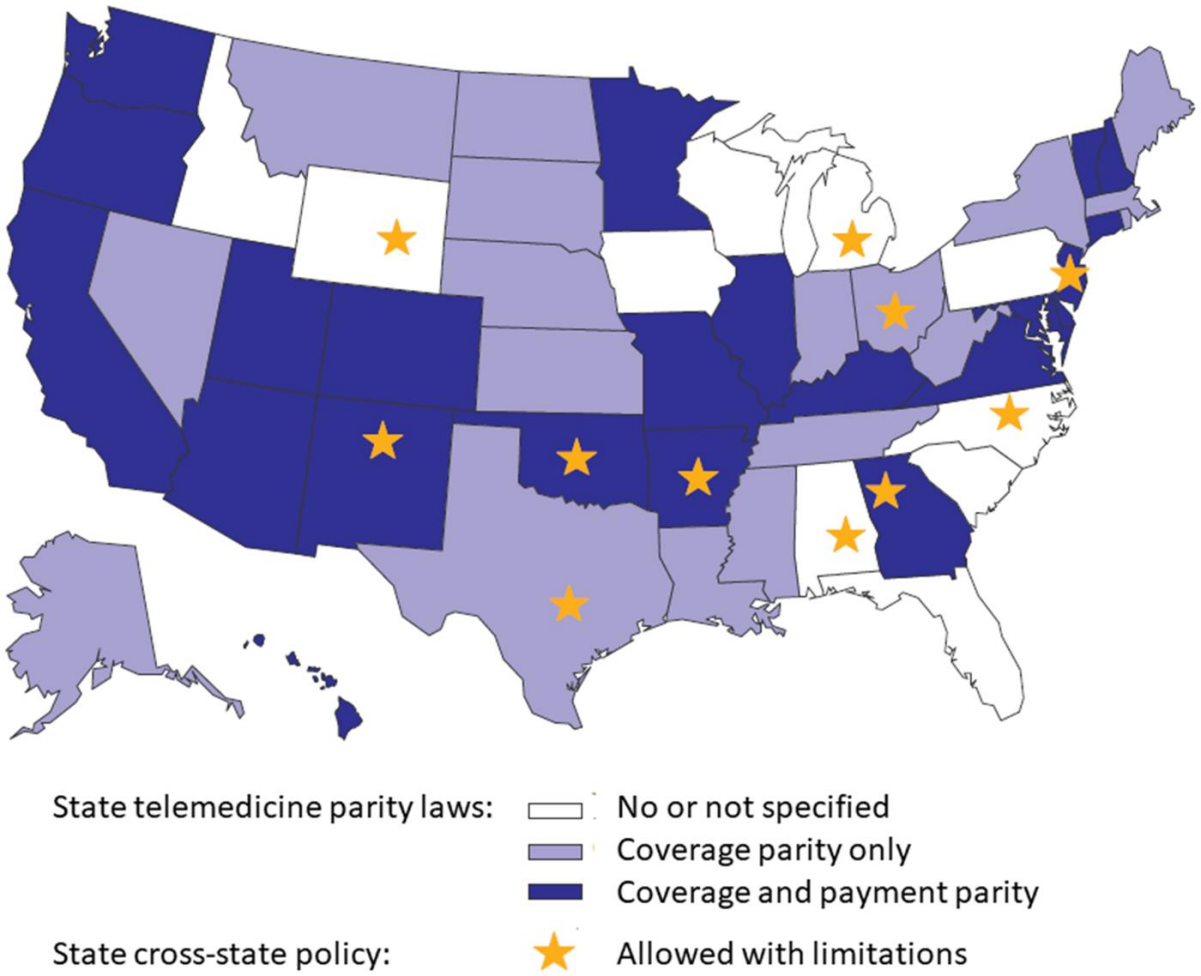
Telemedicine policies, by state. Telemedicine parity law categories: coverage and payment parity (**dark blue**), coverage parity only (**blue**), or no or not specified telemedicine policy (**white**). Cross-state policy categories: allowed (**no star**) or allowed with limitations (**star**).

We categorized patients into 4 subgroups (Supplementary Table 3, available online) based on state of residence and insurance: 1 for patients with a Medicare plan and 3 for those with private insurance by state telehealth parity status (coverage and payment parity [n = 22], coverage parity only [n = 19], and no or no specific telehealth parity law [n = 9]). State policies for telehealth across state lines were dichotomized as allowed vs allowed with limitations (n = 11). Limitations included a temporary special license approval process (n = 8), requirement of a reciprocal or bordering state license (Arkansas, Ohio), or limited time period for telehealth waiver (Michigan). Figure 1 displays each state’s status by parity law and cross-state policy.

### Other variables

Other variables included in the analysis were age at diagnosis, race and ethnicity (Hispanic, non-Hispanic Asian, non-Hispanic Black, non-Hispanic White, and unknown), cancer type, and the month and year of cancer diagnosis.

### Statistical analysis

We used an interrupted time-series analysis to examine the trend in telehealth use relative to the timing of the COVID-19 public health emergency. We also compared characteristics of telehealth users between patients diagnosed with cancer in April to June 2020 (2Q2020) vs January to March 2021 (1Q2021). We conducted multivariable logistic regression to examine factors associated with telehealth use in the first year of the emergency.

We conducted statistical analyses using SAS, version 9.4, statistical software (SAS Institute Inc, Cary, NC) and Stata, release 17.0 (StataCorp LLC, College Station, TX). All statistical tests were 2-sided, and a *P* value of less than .05 was considered statistically significant.

### Sensitivity analysis

Although state laws on cross-state practice apply to all patients, state policies on telehealth parity apply only to fully insured private health plans. Therefore, we conducted sensitivity analyses for the subgroup of privately insured patients younger than 65 years of age.

## Results

### Trend of telehealth use

Of 110 461 patients, the average crude rate of telehealth use within 1 month of cancer diagnosis was 0.3% before the public health emergency (March 2019 to February 2020) and 17.7% between March 2020 and March 2021. The peak rate was 33.6% in April 2020, continually decreased to 12.8% in September 2020, then stayed in the range of 12% to 16% (Figure 2, A). The interrupted time-series analysis (Figure 2, B) showed that the public health emergency statistically significantly affected telehealth use (25% increase; *P *<* *.001). Figure 2, C and D illustrate telehealth use after the emergency by state parity laws and insurance type and by cross-state policies, respectively. By parity law, the highest use was among patients in states with payment and coverage parity. By cross-state policy, telehealth use was higher in states that allowed cross-state coverage without limitations. For the entire cohort and the subgroup of privately insured patients younger than 65 years of age, patients in states with more generous parity status were more likely to have a less restrictive cross-state policy (Table 1, Supplementary Table 4, available online).

**Figure 2.**
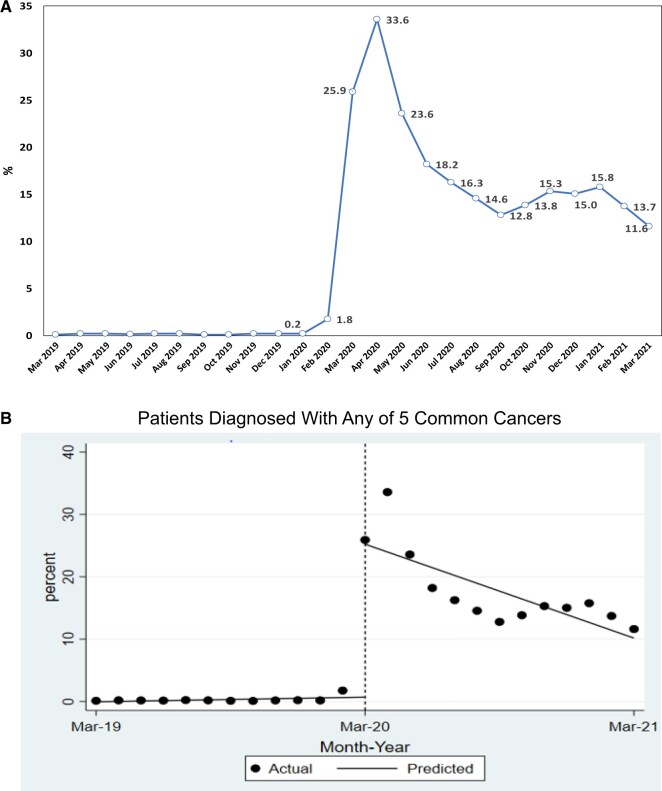
**A)** Trend in telemedicine use in 5 common cancers, March 2019-March 2021. **B)** Results from the interrupted time-series analysis. **C)** Trend in telemedicine use in 5 common cancers, March 2020-March 2021, by state parity law and insurance type. **D)** Trend of telemedicine use in 5 common cancers, March 2020-March 2021, by cross-state policy.

**Figure 2.**
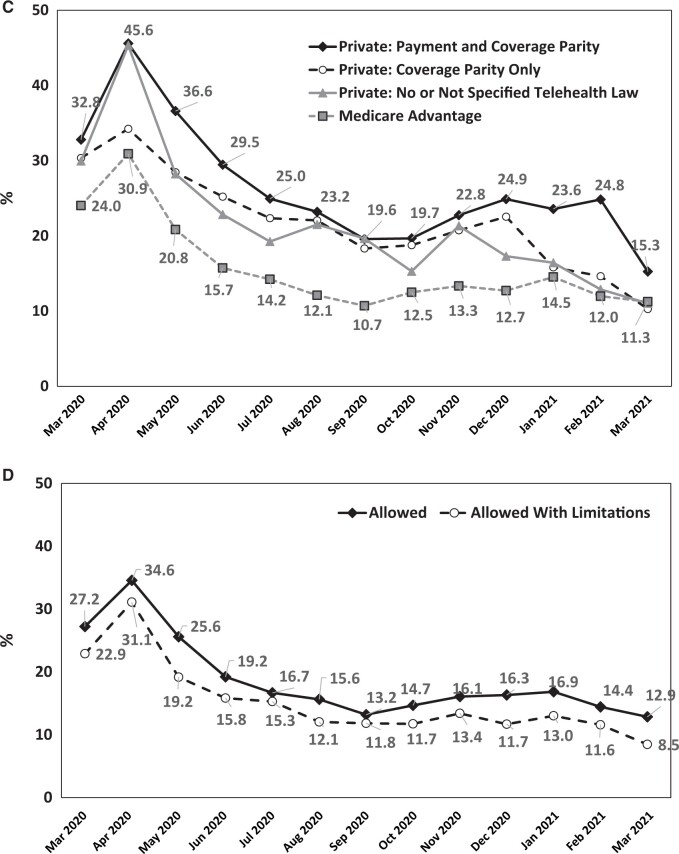
Continued.

**Table 1.** Characteristics of patients with cancer diagnosed between March 2020 and March 2021, total and by cross-state policy

	Total cohort			Cross-state practice allowed			Cross-state practice allowed with limitations			*P* ^a^
Patient characteristics	No.	%	Patients who used telehealth, %	No.	%	Patients who used telehealth, %	No.	%	Patients who used telehealth, %	
Total	53 982	—	17.0	38 125	—	17.94	15 857	—	14.63	<.001
**Age, y**										<.001
20-49	3193	5.9	27.0	2223	5.83	28.43	970	6.12	23.61	
50-64	10 078	18.7	20.4	7014	18.40	21.80	3064	19.32	17.13	
65-74	22 443	41.6	15.9	15 699	41.18	16.92	6744	42.53	13.46	
≥75	18 268	33.8	14.7	13 189	34.59	15.32	6744	32.03	12.96	
**Race and ethnicity**										<.001
Hispanic	4367	8.1	19.2	2832	7.43	20.80	1535	9.68	16.16	
Non-Hispanic Asian	1404	2.6	21.3	1061	2.7	22.62	343	2.16	17.20	
Non-Hispanic Black	6241	11.6	14.1	3187	8.36	15.81	3054	19.26	12.21	
Non-Hispanic White	38 304	71.0	17.2	28 546	74.87	17.91	9758	61.54	15.14	
Unknown	3666	6.8	15.2	2499	6.55	15.73	1167	7.36	13.97	
**Cancer type**										<.001
Breast	17 999	33.3	16.5	12 605	33.06	17.37	5394	34.02	14.48	
Prostate	15 584	28.9	17.2	11 187	29.34	18.46	4397	27.73	14.12	
Lung	9066	16.8	18.7	6345	16.64	19.57	2721	17.16	16.65	
Colorectal	6649	12.3	14.6	4575	12.00	15.54	2074	13.08	12.54	
Lymphoma	4684	8.7	17.8	3413	8.95	18.46	1271	8.02	16.13	
**State telehealth parity laws and insurance type**										
Private insurance plan										<.001
Coverage and payment parity	5953	11.0	25.4	4933	12.94	26.94	1020	6.43	18.14	
Coverage parity only	4003	7.4	21.1	2188	5.74	20.43	1815	11.45	21.98	
No telehealth law or law not specified	2939	5.4	20.9	2232	5.85	20.74	707	4.46	21.22	
Medicare Advantage plan	41 087	76.1	15.1	28 772	75.47	15.98	12 315	77.66	12.88	
**Cross-state telehealth policy**										<.001
Allowed	38 125	70.6	17.9	38 125	100.00	17.9	—	—	—	
Allowed with limitations	15 857	29.4	14.6	—	—	—	15 857	100.00	14.63	
**Census region**										<.001
Northeast	7180	13.30	23.84	6001	15.74	24.48	1179	7.44	20.61	
Midwest	12 619	23.38	15.49	10 616	27.85	15.41	2003	12.63	15.93	
South	22 766	42.17	14.58	10 381	27.23	15.64	12 385	78.10	13.69	
West	11 417	21.15	19.02	11 127	29.19	18.95	290	1.83	21.38	
**Month and year of cancer diagnosis**										<.001
March 2020	3644	6.8	25.9	2535	6.65	27.22	1109	6.99	22.90	
April 2020	2722	5.0	33.6	1944	5.10	34.57	778	4.91	31.11	
May 2020	3428	6.4	23.6	2348	6.16	25.60	1080	6.81	19.17	
June 2020	4556	8.4	18.2	3173	8.32	19.22	1383	8.72	15.84	
July 2020	4651	8.6	16.3	3238	8.49	16.68	1413	8.91	15.29	
August 2020	4707	8.7	14.6	3280	8.60	15.64	1427	9.00	12.05	
September 2020	4870	9.0	12.8	3348	8.78	13.20	1522	9.60	11.83	
October 2020	4606	8.5	13.8	3277	8.60	14.68	1329	8.38	11.74	
November 2020	4001	7.4	15.3	2830	7.42	16.08	1171	7.38	13.41	
December 2020	4145	7.7	15.0	2982	7.82	16.33	1163	7.33	11.69	
January 2021	4210	7.8	15.8	3027	7.94	16.85	1183	7.46	13.02	
February 2021	3998	7.4	13.7	2963	7.77	14.44	1035	6.53	11.59	
March 2021	4444	8.2	11.6	3180	8.34	12.86	1264	7.97	8.47	

a *P* value from χ^2^ tests that compared patient characteristics by cross-state policies.

### Patients characteristics in the postexpansion period

A total of 53 982 patients were diagnosed with 5 common cancers (33.3% with breast cancer, 28.9% with prostate cancer, 16.8% with lung cancer, 12.3% with colorectal cancer, and 8.7% with lymphoma) between March 2020 and March 2021 (Table 1). The majority of patients (71%) were non-Hispanic White; 11.6% were non-Hispanic Black, 8.1% were Hispanic, and 2.6% were Asian. Overall, 42% lived in the southern United States, 23% lived in the Midwest, 21% lived in the western United States, and 13% lived in the northeast United States. More than three-quarters of patients (76.1%) had Medicare Advantage plans, 11.0% had private insurance and resided in a state with telehealth coverage and payment parity, 7.4% lived in a state with coverage parity only, and 5.4% lived in a state with no or unspecified telehealth parity laws. Most (70.6%) patients lived in states that allowed cross-state telehealth; 29.4% resided in states with cross-state policy limitations.

Multivariable logistic regression analysis (Table 2) showed that patients 50 years of age or older were less likely to use telehealth (adjusted rate = 15.7%-17.4% vs 23% for patients aged 20-49 years). Compared with non-Hispanic White patients (adjusted rate = 17.1%), the adjusted rate of telehealth use was lower for non-Hispanic Black patients at 14.7% and higher for non-Hispanic Asian and Hispanic patients at 20.0% and 19.5%, respectively. Compared with those diagnosed with breast cancer (adjusted rates = 15.8%), patients with colorectal cancer were less likely to use telehealth at 14.5%, while those with lung or prostate cancer were more likely to use telehealth at 19.8% and 17.9%, respectively.

**Table 2.** Multivariable logistic regression results of telehealth use within the entire cohort^a^

Patient characteristics	Odds ratio (95% Confidence Interval)	*P*	Adjusted rates of telehealth use
Total (N = 53 982)			17.0
**Age, y**			
20-49	1.00	—	23.0
50-64	0.70 (0.64 to 0.77)	<.001	17.4
65-74	0.67 (0.60 to 0.74)	<.001	16.8
≥75	0.61 (0.55 to 0.69)	<.001	15.7
**Race and ethnicity**			
Hispanic	1.18 (1.08 to 1.28)	<.001	19.5
Non-Hispanic Asian	1.22 (1.07 to 1.41)	.004	20.0
Non-Hispanic Black	0.83 (0.76 to 0.91)	<.001	14.7
Non-Hispanic White	1.00	—	17.1
Unknown	0.86 (0.79 to 0.94)	.001	15.1
**Cancer type**			
Breast	1.00	—	15.8
Prostate	1.17 (1.10 to 1.24)	<.001	17.9
Lung	1.32 (1.24 to 1.42)	<.001	19.8
Colorectal	0.90 (0.84 to 0.97)	.007	14.5
Lymphoma	1.07 (0.99 to 1.16)	.087	16.8
**State telehealth parity laws and insurance type**			
Private Insurance plan			
Coverage and payment parity	1.00	—	23.3
Coverage parity only	0.82 (0.74 to 0.92)	.001	20.2
No telehealth law or law not specified	0.77 (0.70 to 0.85)	<.001	19.1
Medicare Advantage plan	0.59 (0.54 to 0.65)	<.001	15.5
**Cross-state telehealth policy**			
Allowed	1.00	—	17.8
Allowed with limitations	0.80 (0.76 to 0.85)	<.001	14.9
**Month and year of cancer diagnosis**			
March 2020	1.00	—	25.7
April 2020	1.46 (1.30 to 1.63)	<.001	33.4
May 2020	0.88 (0.79 to 0.99)	.032	23.5
June 2020	0.64 (0.58 to 0.71)	<.001	18.3
July 2020	0.56 (0.50 to 0.63)	<.001	16.3
August 2020	0.49 (0.44 to 0.54)	<.001	14.6
September 2020	0.42 (0.37 to 0.47)	<.001	12.8
October 2020	0.46 (0.41 to 0.52)	<.001	13.9
November 2020	0.52 (0.46 to 0.59)	<.001	15.3
December 2020	0.50 (0.45 to 0.56)	<.001	15.0
January 2021	0.53 (0.48 to 0.59)	<.001	15.7
February 2021	0.45 (0.39 to 0.51)	<.001	13.6
March 2021	0.37 (0.33 to 0.41)	<.001	11.5

a Census region variables were not included in multivariable analysis because of collinearity with variables characterizing state polices.

Compared with privately insured patients who lived in states with coverage and payment parity (adjusted rates = 23.2%), those who had private insurance but resided in states with coverage parity only or with no or unspecified telehealth parity law were less likely to use telehealth had adjusted rates of 20.2% and 19.1%, respectively. Patients with Medicare Advantage were least likely to use telehealth (adjusted rate = 15.5%). Patients residing in states with cross-state telehealth policy limitations had lower rates of telehealth use than those in states with cross-state telehealth policies without restrictions (adjusted rate = 14.9% vs 17.8%).

A comparison of telehealth users in 2Q2020 vs 1Q2021 showed a higher proportion of users in 1Q2021 residing in states with coverage and payment parity and in states with no restrictions on cross-state policy (Supplementary Table 5, available online).

### Sensitivity analysis

For the subgroup of 10 813 patients who had private insurance and were younger than 65 years of age, the adjusted rate of telehealth use was higher (23.6%). Factors and patterns associated with telehealth use, however, were similar to those reported in the full study cohort (Table 3).

**Table 3.** Multivariable logistic regression results of telehealth use among patients younger than 65 years of age with private insurance^a^

Patient characteristics	Odds ratio (95% Confidence interval)	*P*	Adjusted rates of telehealth use
Total (N = 10 813)			23.6
**Age, y**			
20-49	1.00	—	28.4
50-64	0.69 (0.62 to 0.77)	<.001	21.7
**Race and ethnicity**			
Hispanic	1.12 (0.96 to 1.31)	.136	25.8
Non-Hispanic Asian	1.13 (0.87 to 1.47)	.353	26.0
Non-Hispanic Black	0.84 (0.72 to 0.98)	.023	20.8
Non-Hispanic White [Referent]	1.00	—	23.7
Unknown	0.91 (0.76 to 1.08)	.265	22.1
**Cancer type**			
Breast	1.00	—	23.6
Prostate	1.12 (1.00 to 1.26)	.058	25.6
Lung	1.19 (0.99 to 1.42)	.064	26.6
Colorectal	0.82 (0.70 to 0.96)	.013	20.3
Lymphoma	0.91 (0.76 to 1.09)	.311	22.0
**State telehealth parity laws and insurance type**			
Private Insurance plan			
Coverage and payment parity	1.00	—	25.9
Coverage parity only	0.79 (0.71 to 0.88)	.001	21.8
No telehealth law or law not specified	0.77 (0.68 to 0.87)	<.001	21.3
**Cross-state telehealth policy**			
Allowed	1.00	—	24.4
Allowed with limitations	0.84 (0.76 to 0.93)	.001	21.4
**Month and year of cancer diagnosis**			
March 2020	1.00	—	32.4
April 2020	1.55 (1.25 to 1.93)	<.001	42.5
May 2020	1.03 (0.83 to 1.28)	.795	33.0
June 2020	0.77 (0.63 to 0.95)	.015	27.1
July 2020	0.63 (0.52 to 0.78)	<.001	23.4
August 2020	0.61 (0.50 to 0.74)	<.001	22.6
September 2020	0.53 (0.43 to 0.66)	<.001	20.3
October 2020	0.48 (0.39 to 0.60)	<.001	18.9
November 2020	0.61 (0.49 to 0.76)	<.001	22.8
December 2020	0.58 (0.47 to 0.73)	<.001	22.0
January 2021	0.54 (0.43 to 0.66)	<.001	20.5
February 2021	0.51 (0.42 to 0.62)	<.001	19.8
March 2021	0.29 (0.23 to 0.37)	<.001	12.5

a Census region variables were not included in multivariable analysis because of collinearity with variables characterizing state polices.

## Discussion

In this cohort of more than 53 000 patients newly diagnosed with the 4 most common solid tumors (breast, lung, colorectal, and prostate) or lymphoma during the first year of the COVID-19 public health emergency, we found that telehealth use was associated with the generosity of parity laws and the restrictiveness of rules governing cross-state practices, and the impact of state policies appeared to be amplified over time based on the comparison of telehealth user characteristics between 2Q2020 and 1Q2021. Overall, telehealth use within 1 month of cancer diagnosis peaked in April 2020, plateaued in September 2020, then leveled off to 12% to 16%. This trend confirms that seen in prior studies in both general (13,14,23,24) and cancer-specific populations (11,12). Similar to prior studies (11,14,23,24), we demonstrated a slight increase in telemedicine use between November 2020 and January 2021, presumably resulting from the surge in postholiday cases. Telehealth use was higher in patients who were younger, Asian, or Hispanic or had lung or prostate cancer and lower in patients who were non-Hispanic Black or had colorectal cancer. Our finding that patients with cancer who are older (12,25,26) and Black (26,27) are less likely to use telehealth are consistent with prior studies. Higher use among Asian and Hispanic patients has not previously been demonstrated in the oncology population; prior smaller, single-institution studies have reported no association with being Asian or Hispanic (26,27).

Most importantly, our study concluded that telehealth use was sensitive to state policies, some affecting only the privately insured. Among the privately insured, the rate of telehealth use among those who lived in states with coverage parity only or unspecified parity policies was 14% to 18% lower than among those in states with both coverage and payment parity. Although state parity laws were relaxed with temporary provisions or waivers since March 2020, we did not have reliable access to the effective duration of all the temporary changes during the study period and therefore categorized states based on the policies that were in effect before the public health emergency. Despite biases toward no difference, we demonstrated that telehealth use during the first year of the pandemic was highest among the privately insured patients who lived in states with prepandemic regulations supporting coverage and payment parity. Our findings support the importance of payment parity for telehealth services.

During the study period, health-care professional licensure regulations regarding cross-state telehealth were temporarily waived to varying degrees. All states and the District of Columbia allowed cross-state telehealth, but 11 had limitations or additional requirements. Telehealth use for patients living in states with additional requirements was more than 15% lower than for those in states without restrictions (adjusted rate = 17.8% vs 14.9%).

Our study is the first to examine the effect of state regulations governing out-of-state telehealth use during the COVID-19 public health emergency. Two studies have examined the receipt of out-of-state telehealth care among Medicare fee-for-service beneficiaries during the COVID-19 pandemic (24,28). Both found that 5% of telehealth visits were by out-of-state clinicians, and most out-of-state telehealth visits occurred between a patient and clinician in a bordering or adjacent state and were for ongoing care with an established clinician. For specialty care, the use of out-of-state telehealth was highest for cancer care (9.8%), followed by hematology care (6.5%) (24). These studies showed that although the use of out-of-state telehealth may be relatively small, it is an important option for continuity of care, particularly for patients living in areas facing specialty care shortages and access issues, including oncology care. Our study highlights that requiring additional processes to allow health-care professionals to practice across state lines is associated with decreased telehealth use.

We found the rate of telehealth use among patients with Medicare Advantage was approximately 20% to 30% lower than among those with private insurance, despite having insurance coverage for these services. This lower use may be attributed to reasons such as patient and clinician preferences, complexity of care, digital literacy/comfort with technology, access to digital technology, and reliable internet coverage (2,11,12,29,30).

On December 16, 2022, only 8 states had public health emergency declarations in place (15). As of May 24, 2023, only 2 states maintained licensure flexibilities, and 21 states have long-term or permanent interstate telehealth policies in place, with varying mechanisms and requirements (eg, telehealth or special license or permit, registration or waiver) (22). As state governments consider updating their prepandemic telehealth policies, major issues under consideration include permanent expansion of services, coverage and payment parity and/or equity for services, removing health-care professional and patient limitations on where services can be accessed (eg, patient home), use of audio-only visits, and investing in appropriate infrastructure and broadband access in areas with fewer resources (2,3,11,27,30).

In addition, health-care professional licensing regulations as well as malpractice and liability insurance for interstate telehealth services must be revisited (2,3,11,24,30,31). More than three-quarters of health-care professionals are licensed to practice in only 1 state (32). Streamlining the administrative burden of obtaining multiple state licenses is imperative. The interstate medical licensure compact is 1 option, but it does not address the costs of maintaining multiple state licenses, and few clinicians (<0.5%) exercise this option (7,31). A more efficient approach would be licensure reciprocity agreements, particularly between adjacent states (31,33). These reciprocity agreements, however, would require state legislatures to adopt licensure compacts or other forms of recognition across state boards.

The American Medical Association promotes increased portability of care without compromising patient safety, advocating that health-care professionals delivering telehealth services be licensed in the state in which the patient receives care and encouraging states to facilitate interstate telehealth services for patients who have an established clinician to allow continuity of care (34). The Federation of State Medical Boards adopted a policy in April 2022 that permits patients who were temporarily located in a different state from their established clinician to continue receiving care by telehealth (35). At least 2 state boards have adopted this policy (32,36).

The American Society of Clinical Oncology’s 2021 position statement similarly recommends that Centers for Medicare & Medicaid Services maintain the expanded telehealth policies that were implemented during the public health emergency and that policies permitting cross-state telehealth require an established patient-clinician relationship (37). Given the long-term relationships patients establish with their oncology clinicians and the often chronic nature of management of cancer and treatment sequelae, telehealth is an excellent modality to incorporate into oncology care delivery models. Uncertainty exists, however, as to the optimal use of telehealth for oncology care because information regarding ideal patient scenarios, care outcomes and safety, and patient and clinician satisfaction is largely unavailable (29,38,39).

Our study has several limitations. The study cohort consisted of patients enrolled in a large insurance company (UnitedHealthcare). Although these patients were diverse with respect to age, race and ethnicity, and geographic representation, the telehealth utilization pattern may not be generalizable to patients with other insurers. As our study focused on state policies, we did not include broadband/Wi-Fi access because access likely varies more widely across counties but less so across states. State parity status was based on policies in effect before or during the public health emergency. We acknowledge that temporary changes to broaden state parity policies occurred during the 1-year time frame of the study, but specific information about and dates for temporary policy changes are not available for every state. Misclassification of out-of-state telehealth policies that were in effect during the study period is possible. Finally, examining telehealth outcomes and how telehealth was used in the context of other patient care was beyond the scope of this study.

The COVID-19 public health emergency ended in May 2023, and federal and state governments are deciding whether to relax certain telehealth polies permanently or return to more restrictive prepandemic policies. Our analysis of telehealth use during the early part of the pandemic suggests that patients residing in states that move toward less generous parity and more restrictive cross-state practice rules may be less likely to benefit from telehealth.

## Supplementary Material

pkad072_Supplementary_Data

## Data Availability

The raw and processed data required to reproduce these findings cannot be shared under the data use agreement between The University of Texas MD Anderson Cancer Center and Optum Inc.
