# Supplementary material for: Impact of state telehealth policies on telehealth use among patients with newly diagnosed cancer
Source: JNCI Cancer Spectr. 2023 Sep 15;7(5):pkad072. doi: 10.1093/jncics/pkad072 (PMC10597585; doi:10.1093/jncics/pkad072)
Supplement: pkad072_Supplementary_Data [file pkad072_supplementary_data.pdf]

SUPPLEMENTARY MATERIALS

Supplementary Table 1. Inclusion and Exclusion Criteria for Cohort Ascertainment

Description	Total	Breast Cancer	Prostate Cancer	Colorectal Cancer	Lung Cancer	Lymphoma
Patients with any of 5 common cancers diagnosed between 3/1/2019 and 3/31/2021		123 767	125 169	47 960	61 137	38 364
Patients had 3 cancer claims on separated dates within three months after the first date of cancer diagnosis (index date)		60 315	56 215	27 550	41 076	19 887
Full enrollment 3 months before and after index date of cancer diagnosis (6 months full enrollment)		42 029	40 334	18 766	25 922	12 924
Excluded cases with any cancer diagnosis within 3 months prior to index date of cancer diagnosis		36 990	31 921	13 636	18 723	9485
Cohort 1 for interrupt time series analysis to analyze the trend of telehealth utilization. Included patients with cancer diagnosis between March 2019 and March 2021. Excluded patients with missing state or age information.	**110 461**	36 890	31 847	13 588	18 687	9449
Cohort 2 for multivariable logistic regression analysis to investigate the factors associated with the use of telehealth. Included patients with cancer diagnosis between March 2020 and March 2021. Excluded patients with missing state or age information.	**53 982**	17 999	15 584	6649	9066	4684

Supplementary Table 2: Codes Used for Cancer Type and Telehealth

**Cancer type**	**ICD-10 Code**
Breast (Female)	C5001, C5011, C5021, C5031, C5041, C5051, C5061, C5071, C5081, C5091
Prostate	C61XXXX
Colorectal	C18XXXX, C19XXXX, C20XXXX
Lung	C341XXX, C342XXX, C343XXX, C348XXX, C349XXX
Lymphoma	C81-C88
**Types of telehealth[1-6]**	**HCPCS/CPT**
Telehealth visits	G0406-G0408, G0425-G0427, G0508-G0509
E-visits	98969, 98970-98972, 99421-99423, 99444, 99446-99449, G2061-G2063
Virtual check in	G0071, G2010, G2012, G2250, G2251
Audio/Telephone only services	98966-98968, 99441-99443
Others	99451-99452, G2025, Q3014, T1014
	Other temporary HCPCS codes for Public Health Emergency for the COVID-19 pandemic with a telehealth modifier 95, GT, or GQ or with a place of services code '02'.

CPT: current procedural terminology; HCPCS: healthcare common procedure coding system.

1. Ellison J, Cole MB, Thompson TA. Association of Telehealth Reimbursement Parity With Contraceptive Visits During the COVID-19 Pandemic. *JAMA Netw Open.* 2022;5(4):e226732.
2. Jones CM, Shoff C, Hodges K, et al. Receipt of Telehealth Services, Receipt and Retention of Medications for Opioid Use Disorder, and Medically Treated Overdose Among Medicare Beneficiaries Before and During the COVID-19 Pandemic. *JAMA Psychiatry.* 2022;79(10):981-992.
3. Katz AJ, Haynes K, Du S, Barron J, Kubik R, Chen RC. Evaluation of Telemedicine Use Among US Patients With Newly Diagnosed Cancer by Socioeconomic Status. *JAMA Oncol.* 2022;8(1):161-163.
4. Mehrotra A, Huskamp HA, Nimgaonkar A, Chaiyachati KH, Bressman E, Richman B. Receipt of Out-of-State Telemedicine Visits Among Medicare Beneficiaries During the COVID-19 Pandemic. *JAMA Health Forum.* 2022;3(9):e223013.
5. Centers for Medicare & Medicaid Services (CMS). List of Telehealth Services Centers for Medicare & Medicaid Services. https://www.cms.gov/Medicare/Medicare-General-Information/Telehealth/Telehealth-Codes. May 9, 2023. Accessed August 11, 2023.

6. American Medical Association (AMA). Telehealth Services Covered by Medicare and Included in CPT Code Set . American Medical Association https://www.ama-assn.org/system/files/telehealth-services-covered-by-medicare-and-included-in-cpt-code-set.pdf. May 1, 2020. Accessed August 11, 2023.

Supplementary Table 3: State policies for coverage and payment parity and cross-state telehealth

State	Telehealth private insurance parity law status[1]	Cross-State Policy[2]	State executive order or other announcement during COVID pandemic related to cross-state policy for telemedicine
**NORTHEAST**			
Connecticut	coverage and payment parity	Allowed	https://www.cga.ct.gov/current/pub/chap_370.htm#sec_20-12
New Hampshire	coverage and payment parity	Allowed	https://www.governor.nh.gov/sites/g/files/ehbemt336/files/documents/emergency-order-15.pdf
Vermont	coverage and payment parity	Allowed	https://legislature.vermont.gov/Documents/2022/Docs/ACTS/ACT085/ACT085%20As%20Enacted.pdf
New Jersey	coverage and payment parity	Limited	https://www.nj.gov/oag/newsreleases20/pr20200320a.html
Maine	coverage parity only	Allowed	https://www.maine.gov/md/sites/maine.gov.md/files/inline-files/Medical%20Licensing%20and%20Telehealth%20Executive%20Order.pdf
Massachusetts	coverage parity only	Allowed	https://www.mass.gov/doc/order-maximizing-available-healthcare-providers-april-2020/download
New York	coverage parity only	Allowed	No information available online describing policy in effect during the study period.
Rhode Island	coverage parity only	Allowed	https://health.ri.gov/publications/policies/RIDOH-Policies-Telemedicine.BMLD.3.20.20.pdf
Pennsylvania	No or not specified telemedicine law	Allowed	https://www.dhs.pa.gov/providers/Providers/Documents/Coronavirus%202020/QTip%20242%20Reissued%204-20-2020.pdf
**MIDWEST**			
Illinois	coverage and payment parity	Allowed	https://coronavirus.illinois.gov/content/dam/soi/en/web/coronavirus/documents/2020%2003%2030%20guidance%20for%20healthcare%20providers%20using%20telehealth%20services%20in%20illinois.pdf
Minnesota	coverage and payment parity	Allowed	https://mn.gov/boards/assets/EO%2020-46%20Out%20of%20State%20Heatlhcare%20Workers%20-%20FINAL%20-%2004272020_tcm21-429904.pdf
Missouri	coverage and payment parity	Allowed	https://standard-democrat.com/story/2679203.html
Indiana	coverage parity only	Allowed	https://www.in.gov/gov/files/Executive%20Order%2020-13%20Medical%20Surge.pdf<br>https://www.in.gov/gov/files/EO_20-05.pdf

Iowa	coverage parity only	Allowed	https://governor.iowa.gov/sites/default/files/documents/Public%20Health%20Proclamation%20-%202020.11.10.pdf
Kansas	coverage parity only	Allowed	https://governor.kansas.gov/wp-content/uploads/2020/03/E.O.-20-08.pdf
Nebraska	coverage parity only	Allowed	http://govdocs.nebraska.gov/docs/pilot/pubs/eofiles/20-10.pdf
North Dakota	coverage parity only	Allowed	https://www.governor.nd.gov/sites/www/files/documents/executive-orders/Executive%20Order%202020-05.01.pdf
South Dakota	coverage parity only	Allowed	https://sdsos.gov/general-information/executive-actions/executive-orders/assets/2020-07.PDF
Ohio	coverage parity only	Limited	https://clt945532.bmeurl.co/A299F0A
Wisconsin	No or not specified telemedicine law	Allowed	https://evers.wi.gov/Documents/COVID19/EMO16-DSPSCredentialingHealthCareProviders.pdf
Michigan	No or not specified telemedicine law	Limited	https://content.govdelivery.com/attachments/MIEOG/2020/05/14/file_attachments/1451862/EO%2020 20-86%20Emerg%20order%20-%20telehealth.pdf https://www.michigan.gov/whitmer/news/state-orders-and-directives/2020/04/27/executive-order-2020-61
**SOUTH**			
Delaware	coverage and payment parity	Allowed	https://news.delaware.gov/2020/03/23/governor-carney-declares-public-health-emergency-phe/
Kentucky	coverage and payment parity	Allowed	https://chfs.ky.gov/agencies/ohda/telehealth/COVID19TelehealthGuidance.docx
Maryland	coverage and payment parity	Allowed	https://mgaleg.maryland.gov/2020RS/Chapters_noln/CH_13_sb1080e.pdf https://www.mbp.state.md.us/forms/TelehealthFAQs.pdf
Virginia	coverage and payment parity	Allowed	https://web.archive.org/web/20210303174922/https:/www.governor.virginia.gov/media/governorvirginiagov/executive-actions/EO-57-Licensing-of-Health-Care-Professionals-in-Response-to-Novel-Coronavirus-(COVID-19).pdf
Arkansas	coverage and payment parity	Limited	https://www.armedicalboard.org/Professionals/pdf/Website%20COVID-19%20Border%20State%20Emergency%20Temporary%20License%20MD%20DO%20050120.pdf
Georgia	coverage and payment parity	Limited	https://law.justia.com/codes/georgia/2020/title-43/chapter-34/article-2/section-43-34-31/
Oklahoma	coverage and payment parity	Limited	https://www.sos.ok.gov/documents/executive/1914.pdf

District of Columbia	coverage parity only	Allowed	https://dchealth.dc.gov/sites/default/files/dc/sites/doh/page_content/attachments/Order%20-%20Licensure%20Waivers.20.03.13.pdf
Louisiana	coverage parity only	Allowed	https://gov.louisiana.gov/assets/Proclamations/2022/18JBE2022StateofEmergencyCOVID.pdf
Mississippi	coverage parity only	Allowed	https://www.msbml.ms.gov/sites/default/files/news/Supplemental_Proclamation.pdf
Tennessee	coverage parity only	Allowed	https://www.jdsupra.com/legalnews/gov-bill-lee-enacts-significant-14113/
West Virginia	coverage parity only	Allowed	https://apps.sos.wv.gov/adlaw/executivejournal/readpdf.aspx?DocID=89501
Texas	coverage parity only	Limited	https://www.akerman.com/en/perspectives/texas-telemedicine-updates.html
Florida	No or not specified telemedicine law	Allowed	https://www.flhealthsource.gov/pdf/emergencyorder-20-002.pdf?inf_contact_key=c1be7c474d297aa416752a23d2694901680f8914173f9191b1c0223e68310bb1
South Carolina	No or not specified telemedicine law	Allowed	https://www.jdsupra.com/legalnews/south-carolina-healthcare-agency-76094/?
Alabama	No or not specified telemedicine law	Limited	https://www.commerce.alaska.gov/web/Portals/5/pub/TelehealthLicensingGuidelinesCOVID.pdf
North Carolina	No or not specified telemedicine law	Limited	https://files.nc.gov/governor/documents/files/EO130-Meeting-North-Carolinas-Health-and-Human-Services-Needs.pdf
**WEST**			
Arizona	coverage and payment parity	Allowed	https://www.azdhs.gov/covid19/documents/adhs-admin-order-2020-01.pdf
California	coverage and payment parity	Allowed	https://www.gov.ca.gov/wp-content/uploads/2020/03/3.4.20-Coronavirus-SOE-Proclamation.pdf
Colorado	coverage and payment parity	Allowed	https://docs.google.com/document/d/1fqo3qm8QQZG5vnzYEKg0BA1FgEBH-rBpRnPy0WeCDTw/edit
Hawaii	coverage and payment parity	Allowed	https://governor.hawaii.gov/wp-content/uploads/2021/06/2106080-ATG_21st-Emergency-Proclamation-for-COVID-19-distribution-signed.pdf
Oregon	coverage and payment parity	Allowed	https://www.phe.gov/Preparedness/legal/prepact/Pages/4-PREP-Act.aspx
Utah	coverage and payment parity	Allowed	No information available online describing policy in effect during the study period.

Washington	coverage and payment parity	Allowed	https://content.govdelivery.com/accounts/WAMC/bulletins/2809de0
New Mexico	coverage and payment parity	Limited	https://www.nmmb.state.nm.us/docs/license_apps/emergency_md/FACT%20SHEET%20and%20FAQs%20for%20FED%20EMERGENCY%20LICENSE%204.2020%20(1).pdf
Alaska	coverage parity	Allowed	https://www.alabamapublichealth.gov/legal/assets/med-ex-540-x-7-.69-er.pdf
Montana	coverage parity only	Allowed	https://web.archive.org/web/20200623092727/https:/boards.bsd.dli.mt.gov/Portals/133/Documents/med/COVID-19/Directive%20on%20Telehealth.pdf?ver=2020-03-24-082744-897
Nevada	coverage parity only	Allowed	https://gov.nv.gov/News/Emergency_Orders/2020/2020-04-01_-_COVID-19_Declaration_of_Emergency_Directive_011/
Idaho	No or not specified telemedicine law	Allowed	https://gov.idaho.gov/wp-content/uploads/2020/03/covid-19-declaration_final.pdf
Wyoming	No or not specified telemedicine law	Limited	https://web.archive.org/web/20210801172600/http:/wyomedboard.wyo.gov/resources/covid-19

[a] Florida and Michigan have private insurance laws in place. Since neither state's laws explicitly require coverage parity, both states were classified in the "no or not specified telehealth law" group.

[b] Cross-state policies in effect during the study period were obtained in 2022 and the source listed. Several state policies have subsequently been removed and are presumably now outdated (AR, CO, HI, IA, KY. NV).

1. National Conference of State Legislatures (NCSL). The Telehealth Explainer Series. Telehealth Private Insurance Laws. National Conference of State Legislatures (NCSL). https://www.ncsl.org/health/the-telehealth-explainer-series/telehealth-private-insurance-laws. July 15, 2021. Accessed August 11, 2023.

2. Federation of State Medical Boards (FSMB). U.S. States and Territories Modifying Requirements for Telehealth in Response to COVID-19. https://www.fsmb.org/siteassets/advocacy/pdf/states-waiving-licensure-requirements-for-telehealth-in-response-to-covid-19.pdf. May 24, 2023. Accessed August 11, 2023.

Supplementary Table 4: Characteristics of Privately Insured Non-Elderly Patients with Cancer Diagnosis Between March 2020 and March 2021, By State Parity Policies

Patient Characteristics	Coverage and Payment Parity		Coverage Parity Only		No or Not Mentioned		P value
	N	%	N	%	N	%	
**Total**	4969		3362		2482		<0.001
**Age (Year)**							0.083
20-49	1401	28.19	997	29.65	671	27.03	
50-64	3568	71.81	2365	70.35	1811	72.97	
65-74	-	-	-	-	-	-	
75+	-	-	-	-	-	-	
**Race and ethnicity**							
Non-Hispanic White	3527	70.98	2404	71.51	1768	71.23	<0.001
Non-Hispanic Black	437	8.79	362	10.77	290	11.68	
Non-Hispanic Asian	249	5.01	112	3.33	47	1.89	
Hispanic	395	7.95	311	9.25	220	8.86	
Unknown	361	7.27	173	5.15	157	6.33	
**Cancer type**							<0.001
Breast	2380	47.90	1544	45.93	1109	44.68	
Prostate	1076	21.65	739	21.98	571	23.01	
Lung	315	6.34	292	8.69	211	8.50	
Colorectal	687	13.83	434	12.91	314	12.65	
Lymphoma	511	10.28	353	10.50	277	11.16	
**State telehealth parity laws and insurance type**							<0.001
Private Insurance plan							
Coverage and payment parity	4969	100%	-	-	-	-	
Coverage parity only	-	-	3362	100%	-	-	
Telehealth law, no or not specified	-	-	-	-	2482	100%	
Medicare Advantage plan	-	-	-	-	-	-	
**Cross state telehealth policy**							<0.001
Allowed	4112	82.75	1822	54.19	1859	74.90	
Allowed with limitations	857	17.25	1540	45.81	623	25.10	
**Census Region**							<0.001
Northeast	290	5.84	537	15.97	214	8.62	
Midwest	1391	27.99	1126	33.49	573	23.09	
South	1380	27.77	1614	48.01	1660	66.88	
West	1908	21.15	85	2.53	35	1.41	

**Month and year of cancer diagnosis**							<0.001
March 2020	357	7.18	233	6.93	187	7.53	
April 2020	247	4.97	148	4.40	132	5.32	
May 2020	315	6.34	209	6.22	183	7.37	
June 2020	394	7.93	289	8.60	195	7.86	
July 2020	438	8.81	285	8.48	174	7.01	
August 2020	420	8.45	289	8.60	238	9.59	
September 2020	466	9.38	332	9.88	191	7.70	
October 2020	382	7.69	266	7.91	205	8.26	
November 2020	343	6.90	263	7.82	183	7.37	
December 2020	393	7.91	258	7.67	203	8.18	
January 2021	402	8.09	252	7.50	172	6.93	
February 2021	390	7.85	233	6.93	207	8.34	
March 2021	422	8.49	305	9.07	212	8.54	

Supplementary Table 5: Comparison of Patient Characteristics of Telehealth Users with Cancer Diagnosis in April 2020 - June 2020 and January 2021 - March 2021

Patient Characteristics	Cancer Diagnosis April 2020 – June 2020		Cancer Diagnosis January 2021 – March 2021		P value
	N	%	N	%	
**Total**	2,666	100%	1,728	100%	
**Age (Year)**					0.023
20-49	212	7.95	166	9.61	
50-64	613	22.99	348	20.14	
65-74	1,004	37.66	694	40.16	
75+	837	31.40	520	30.09	
**Race and ethnicity**					0.082
Non-Hispanic White	1,936	72.62	1,249	72.28	
Non-Hispanic Black	276	10.35	145	8.39	
Non-Hispanic Asian	79	2.96	51	2.95	
Hispanic	216	8.10	169	9.78	
Unknown	159	5.96	114	6.60	
**Cancer type**					0.164
Breast	797	29.89	566	32.75	
Prostate	848	31.81	498	28.82	
Lung	515	19.32	325	18.81	
Colorectal	269	10.09	187	10.82	
Lymphoma	237	8.89	152	8.80	
**State telehealth parity laws and insurance type**					0.017
Private Insurance plan					
Coverage and payment parity	413	15.49	307	17.77	
Coverage parity only	218	8.18	128	7.41	
Telehealth law, no or not specified	197	7.39	94	5.44	
Medicare Advantage plan	1,838	68.94	1,199	69.3	
**Cross state telehealth policy**					0.001
Allowed	1,963	73.63	1,347	77.95	
Allowed with limitations	703	26.37	381	22.05	
**Census Region**					<0.001
Northeast	500	18.75	326	18.87	
Midwest	631	23.67	350	20.25	
South	989	37.10	570	32.99	
West	546	20.48	482	27.89	
